# Cultural and contextual relevance of the Indigenous data in the Canadian longitudinal study on aging

**DOI:** 10.17269/s41997-025-01087-5

**Published:** 2025-08-19

**Authors:** Grace M. Spiro, Megan E. O’Connell, Chaneesa Ryan, Laura Warren, Native Women’s Association of Canada (NWAC), Jennifer D. Walker

**Affiliations:** 1 Institute for Health Policy, Management and Evaluation, University of Toronto, 155 College St 4 Floor, Toronto, ON Canada; 2 Department of Psychology & Health Studies, University of Saskatchewan, 9 Campus Dr, Saskatoon, SK Canada; 3 Native Women’s Association of Canada, 85 Albert St, Ottawa, ON Canada; 4 Women’s College Research Institute, Women’s College Hospital, Toronto, ON Canada; 5 Department of Health Research Methods, Evidence, and Impact, McMaster University, MDCL 3510. 1280 Main Street West, Hamilton, ON Canada

**Keywords:** CLSA, Indigenous-identified data, Data sovereignty, ÉLCV, Données sur l’identité autochtone, Souveraineté des données

## Abstract

**Objectives:**

The CLSA is a national data platform for aging research that used epidemiology-based sampling methods and explicitly excluded people living on First Nations Reserves and other provincial First Nations settlements as possible CLSA participants. As such, the CLSA research approach did not use Indigenous community engagement. Nevertheless, the CLSA sample includes a sizeable subsample of participants who self-identified as First Nations, Métis, or Inuit. This project seeks to describe the self-identified Indigenous subsample of the CLSA from the baseline data collection and interpret that description with the aid of an Elder Advisory Circle.

**Methods:**

We conducted a descriptive analysis of the self-identified Indigenous subsample of the CLSA from the baseline data collection. The analysis was presented to an Elder Advisory Circle for consultation.

**Results:**

The lack of community-engaged approaches to Indigenous research and sampling approaches appears to have resulted in a sociodemographic profile of older Indigenous Peoples that does not match the lived experience of the Elder Advisory Circle and contrasts with other data available on Indigenous Peoples in Canada. We feel the existing CLSA data does not reflect the sociodemographic profile of older Indigenous Peoples.

**Conclusion:**

We use this community consultation process to provide recommendations for the appropriate use of the Indigenous-identified data in the CLSA, and we conclude by recommending great caution when using the data from the Indigenous subsample in the CLSA data.

## Résumé

**Objectifs:**

L’Étude longitudinale canadienne sur le vieillissement (ÉLCV) est une plateforme nationale de données de recherche sur le vieillissement qui utilise des techniques d’échantillonnage fondées sur l’épidémiologie et qui exclut explicitement comme participants possibles les personnes vivant dans les réserves des Premières Nations et dans d’autres peuplements provinciaux de Premières Nations. De ce fait, l’approche de recherche de l’ÉLCV ne fait pas appel à la participation des communautés autochtones. Néanmoins, l’échantillon de l’ÉLCV inclut un sous-ensemble appréciable de participants qui s’identifient comme étant membres des Premières Nations, Métis ou Inuits. Notre projet visait à décrire ce sous-ensemble basé sur la collecte des données de référence et à interpréter cette description avec l’aide d’un cercle consultatif d’aînés.

**Méthodes:**

Nous avons effectué une analyse descriptive du sous-ensemble de personnes s’identifiant comme étant autochtones dans l’ÉLCV basé sur la collecte des données de référence. Nous avons ensuite présenté notre analyse à un cercle consultatif d’aînés.

**Résultats:**

L’absence d’approches de recherche axées sur les communautés autochtones et d’approches d’échantillonnage semble avoir donné un profil sociodémographique des personnes âgées autochtones qui ne correspond pas au vécu du cercle consultatif d’aînés et qui tranche avec les autres données disponibles sur les personnes autochtones au Canada. Nous jugeons que les données existantes de l’ÉLCV ne reflètent pas le profil sociodémographique des personnes âgées autochtones.

**Conclusion:**

Nous avons utilisé ce processus de consultation des communautés pour formuler des recommandations sur l’usage approprié des données sur l’identité autochtone dans l’ÉLCV; en conclusion, nous recommandons d’utiliser avec une extrême prudence les données du sous-ensemble autochtone des données de l’ÉLCV.

## Introduction

The current global population aging has prompted societies to prepare, and critical to this preparation is the collection of data required for leaders and decision-makers to adequately plan and is a key component of the United Nations Decade of Healthy Aging (2021–2030) (WHO) (Keating, 2022). The Canadian Longitudinal Study on Aging is a national longitudinal study of aging in Canada established in 2010 to support research to guide aging and health policy in Canada (P. Raina et al., 2019; P. S. Raina et al., 2009). While First Nations, Métis, and Inuit populations in Canada have, generally, younger age structures than that of Canada as a whole, the proportion of Indigenous people who are over the age of 65 is increasing (Government of Canada, 2017). Within the next two decades, the proportion of Indigenous people (First Nations, Inuit and Métis) who are aged 65 years and older is projected to more than double in size (Government of Canada, 2017). Conceptualizations of successful aging differ across cultures, and those from Indigenous populations are distinct from other groups (Collings, 2001). From an Indigenous standpoint, “successful aging takes place at the intersection of individual, social, and cultural contexts across the life course, and against particular historical, economic, and political backdrops” (Pace & Grenier, 2017, p.250). Older Indigenous populations have distinct needs that remain largely unmet, partially due to a lack of community-informed and community-led research that approaches aging from an Indigenous perspective. To truly understand the unique needs, priorities, and barriers faced by older Indigenous populations in Canada, it is imperative to engage in research that is led and driven by Indigenous communities. This research should integrate the voices of older Indigenous people, draw upon traditional knowledge, and utilize Indigenous and decolonizing methodologies. Though there exists no standard model for decolonizing research methodology, in the context of research, decolonization involves centring concerns and worldviews of those non-Western individuals previously “Other(ed)” (Battiste & Youngblood, 2000; Datta, 2018; Smith, 2021; Thambinathan & Kinsella, 2021). Improved uptake of Indigenous research methodologies, which allow Indigenous peoples and communities themselves to drive their own research agenda and control how the data is used, is crucial to advance this effort (Canadian Alliance, 2019). Until we have this information, we cannot effectively advocate for tailored, culturally safe programs and policies that can contribute to healthy aging and ultimately reconciliation.

Despite the clear need for First Nations–, Inuit-, and Métis-specific information to guide planning and decision-making by Indigenous leaders and organizations, there is a lack of Indigenous aging data infrastructure. In Canada, critical population health information for the assessment and monitoring of the outcomes and risk factors related to acute and chronic disease is often not available (Walter & Suina, 2019). Data that is available is usually of substandard quality, and either do not contain meaningful and respectful ways to count First Nations, Inuit, or Métis identity (e.g., most administrative health services data); do not adequately sample older individuals (e.g., First Nations Regional Health Survey); or are not available to Indigenous institutions to use for planning and decision-making (e.g., most national surveys). Existing health and health systems data are often analyzed using a medicalized, deficit-based approach to understanding health that is largely incompatible with Indigenous perspectives on well-being. Health data collected ***on*** Indigenous Peoples are commonly presented in a manner that emphasizes the “difference, disparity, disadvantage, dysfunction, and deprivation” of Indigenous populations in relation to the non-Indigenous population (Canadian Alliance, 2019; Carjuzaa & Fenimore-Smith, 2010; Smylie & Firestone, 2015; Walter & Suina, 2019). There is concern that the sole use of such “deficit-based” indicators contributes to the marginalization of local Indigenous theories of health, which incorporate for example notions of balance, positivity, well-being, and inter-relationality at a foundational level (Carjuzaa & Fenimore-Smith, 2010; Dufour, 1993; Kirkness & Barnhardt, 1991; Makokis, 2008; Smylie & Firestone, 2015). Continuous use of a deficit-based approach may also have negative implications for Indigenous individuals and communities, as this can perpetuate negative stereotypes and may lead to internalized negativity and racism (Hyett et al., 2019; Kelm, 1998; O’Neil et al., 1998; Smylie & Firestone, 2015). Historically, research — within and beyond health — in Indigenous communities has been developed, executed, and reviewed by non-Indigenous researchers with minimal, if any collaboration with Indigenous people and little regard for Indigenous knowledge and worldviews (Canadian Institutes of Health Research, 2016). These findings are not seen as valid, useful, beneficial, or relevant by many Indigenous communities and may perpetuate health disparities and associated inequities. As a result, Indigenous knowledge, perspectives, and methodologies have been marginalized, and many Indigenous populations view research with some mistrust and suspicion. In order to support health improvement, Indigenous community leaders, health policy makers, and practitioners cannot promote, deliver, and evaluate evidence-based health interventions such as vaccinations, healthy lifestyle programming, and primary care enhancements without access to health research that is led by Indigenous community members and incorporates Indigenous knowledge systems, worldviews, and perspectives (Dahrouge et al., 2012; Smylie & Firestone, 2015). In response to this overwhelming need for culturally safe, appropriate, and relevant First Nations–, Inuit-, and Métis-specific information, several models of data governance have been established. One such model known as OCAP® was developed by First Nations and centres on ownership; control whereby First Nations hold on how the data are collected, used, and disclosed; access, whereby First Nations have access to any data about them; and possession whereby all First Nations data fall within First Nations’ jurisdiction (First Nations Information Governance Centre, 2014). Following the establishment of OCAP®, similar and adapted sets of principles have emerged in other Indigenous groups within Canada, including the recent *National Inuit Strategy on Research*, which establishes Inuit ownership, control, and access with respect to Inuit data and information (Kanatami, 2018).

These limitations have frustrated efforts made by National Indigenous Organizations to apply population health and epidemiological methods to evaluate and monitor the needs and priorities of Indigenous people across Canada as they age. In 2018, the Native Women’s Association of Canada (NWAC) worked with researchers to access the Canadian Longitudinal Study on Aging (CLSA) data to explore the CLSA as a means to fill this gap. As researchers from NWAC and university-based Indigenous and allied researchers, we requested access to data from the CLSA. It is important to note that the CLSA was not originally designed to be an Indigenous-specific cohort and was not co-designed with Indigenous people. The CLSA was conceived as a national platform in the early 2000 s in collaboration with the Canadian Institutes of Health Research (CIHR), CIHR Institute of Aging, and Statistics Canada. The sampling strategy adopted for the CLSA was to resemble Statistics Canada’s CCHS-Healthy Aging Survey, which does not provide a representative sample of Indigenous people in Canada. There was an acknowledgment by CIHR, its relevant institutes, and the CLSA team that the development of an Indigenous aging cohort would require the involvement of Indigenous scholars and Indigenous communities to develop a study design, data collection approach, and platform that is Indigenous led and meets the needs of aging Indigenous populations in Canada. To date, a distinct funding opportunity for an Indigenous aging cohort in Canada has not emerged. Though the CLSA sampling approach and data collection did not consider Indigenous populations specifically, the baseline data collection did include some information regarding Indigenous identity. All respondents to the CLSA had the opportunity to self-identify as Indigenous and to specify First Nations, Inuit, or Métis ethnicity; cultural and racial backgrounds; and languages spoken.

To ensure that this project was informed by the distinct needs and lived experiences of older Indigenous people, the research team organized a 1-day engagement session in Ottawa, Ontario, with Elders to inform the development of research questions that would be used to guide the analysis of CLSA data. This led to the development of five priority research questions: (1) What is the sociodemographic and health profile of the older Indigenous population in Canada? (2) What is the prevalence of multimorbidity and the characteristics of people with multimorbidity? (3) What informal and formal care do aging Indigenous people access and how does this relate to chronic conditions? (4) How are older Indigenous people promoting healthy aging through social interactions and cultural connection? (5) What is the prevalence of falls and associated injuries among Indigenous respondents? Though these questions were prioritized, not all questions could be answered within the scope of the CLSA data.

Following the preliminary analysis of CLSA data, a second 1-day engagement session grounded in ceremony and cultural practices with Elders was held in Eel River Bar First Nation in New Brunswick. In attendance were 12 Elders and four older Indigenous women who had not previously attended the engagement session in Ottawa. Attendees were asked to provide their input on the preliminary CLSA data analysis and to help determine how to best use the data to represent the health needs of older Indigenous people. In this case, older adults and older Indigenous people were defined as those over 65 years of age. This review of the sociodemographic results led to questioning and ultimately, cautioning, of the validity and utility of the entire CLSA Indigenous cohort for funding decisions, planning, and policy making.

A report based on the results of the second engagement session was shared with the leadership team at the CLSA during an in-person meeting in Hamilton, Ontario. The report to CLSA highlighted: the lack of Indigenous data governance in CLSA processes (which is now being addressed by new process for Indigenous data access, implemented in March 2023 by CLSA), data content that may not reflect Indigenous perspectives and experiences, and sampling that is not representative of Indigenous populations. In this paper, we will address the third concern — sampling — by summarizing the analysis presented to the Elders that describes the self-identified Indigenous sample in the CLSA cohort. It is essential to emphasize that the CLSA was not originally designed to create a dedicated Indigenous cohort, but rather to capture a broad spectrum of aging experiences. Consequently, we aim to shed light on the intricacies of this self-identified Indigenous sample, recognizing that it emerged unintentionally within the CLSA’s broader framework. Additionally, this paper will provide recommendations for the appropriate and respectful use of the Indigenous-identified data in the CLSA.

## Methods

### Study design

Using Indigenous methodologies for quantitative methods, we conducted a community-engaged analysis of quantitative data. The CLSA is a national longitudinal study of aging in Canada (P. Raina et al., 2019; P. S. Raina et al., 2009). Baseline data were collected in 2010–2015 with follow-up data then being obtained every 3 years on participants. The aim is to follow those enrolled participants until death or study completion in 20 years or more. Adults between the ages of 45 and 85 at study entry were included. The initial sampling frame used recruitment from a subset of participants in Statistics Canada’s Canadian Community Health Survey-Healthy Aging (CCHS-HA) (P. Raina et al., 2019). In following this sampling frame, the CLSA excluded residents of the Canadian territories and some remote regions, persons on Federal First Nations reserves and other provincial First Nations settlements, full-time members of the Canadian Armed Forces, and institutionalized persons (including long-term care). In addition to these exclusion criteria, participants had to be able to complete the interviews in English or French and be physically and cognitively able to participate on their own (e.g., able to hear, able to answer). Some of these criteria, such as the exclusion of individuals living on First Nation reserves and Inuit residing in Nunavut and the Northwest Territories, restricted the CLSA from collecting data that represents the Indigenous population across Canada. Instead, the CLSA could only recruit and enroll Indigenous individuals residing in other areas, predominantly urban locations. The CLSA did not intend to collect and develop an Indigenous-identified cohort; however, questions related to Indigenous identity were included.

There are two components of the CLSA: (1) a tracking cohort of 21,241 who were recruited from all provinces and (2) a comprehensive cohort of 30,097 who were recruited from within 25 to 50 km of one of 11 data collection sites (DCS) (P. S. Raina et al., 2009) located in 7 provinces. Each of the two cohorts had a distinct sampling approach. The tracking cohort was a stratified sample (age, sex, and province) from the CCHS-Health Aging Cycle study, provincial health registries (excluding on-reserve) and random digit dialing to landline phone numbers. Surveys were completed using a 60-min telephone interview. For the comprehensive, in-person cohort, 11 data collection sites were established across Canada, all located in major urban centres including: Surrey, Victoria, Vancouver, Calgary, Winnipeg, Hamilton, Ottawa, Montreal, Sherbrooke, Halifax, and St. John’s. This cohort sample was recruited from provincial health registries (excluding on-reserve) and random digit dialing to landline phone numbers. The comprehensive cohort received an in-home face-to-face interview combined with physical assessments, blood, and urine samples. At the time of enrollment, the 51,338 participants were between the ages of 45 and 85. Questionnaires were completed using computer-assisted telephone interviews (tracking cohort) or an in-home face-to-face interview that was combined with a visit to the local DCS for further data collection including physical assessments and both blood and urine collection (comprehensive cohort).

### Data analysis

All data were analyzed using SPSS Statistical software version 24 (IBM Corp, 2016). Data were assessed both separately and collectively for the comprehensive cohort (in-person interview and physical exam, *n* = 1128) and the tracking cohort (telephone interview, *n* = 751) for these analyses. In both cohorts, participants were able to self-identify as First Nations, Métis, Inuit or a combination of such. A derived variable was then created to identify participants as either Indigenous or non-Indigenous using the survey questions regarding Indigenous (First Nations, Inuit, Métis) ethnicity and culture.

Using the baseline data, age categories were created from the continuous age variable (45–54, 55–64, 65–74, 75–85). Univariate analyses were conducted to describe the sociodemographic profile of the Indigenous sample within the CLSA. Variables of interest included: Indigenous ethnicity (First Nations, Métis, Inuit, more than one Indigenous ethnicity), sex (male, female), age (45–54, 55–64, 65–74, 75–85), relationship status (Single or never married, married or common-law, widowed, divorced, separated) by sex, religion (Roman Catholic, other Christian, other/multi-religio, no religion), education overall (completed high school, bachelor’s degree or higher, trade certificate or diploma from a vocational school/community college/CEGEP/apprenticeship program) by sex and age category, and household income (< 20,000; 20,000–49,999; 50,000–99,999; 100,000–149,000; > 150,000) by sex. These analyses did not use sampling weights because it is unclear how these weights would apply to Indigenous Peoples (CLSA, 2017).

## Results

In total, 1879 self-identified Indigenous participants were enrolled in the CLSA and underwent a baseline evaluation. This accounted for 3.7% of the total CLSA sample. Of the Indigenous participants included in the study, 60% (*n* = 1128) were enrolled in the comprehensive cohort and 40% (*n* = 751) in the tracking cohort. The Indigenous study sample included 72% (*n* = 1360) self-identified First Nations, 29% (*n* = 550) self-identified Métis, 3% (*n* = 58) self-identified Inuit, and 5% (*n* = 88) participants listing more than one Indigenous identity. A complete description of sociodemographic information for each of the two sampling cohorts (comprehensive and tracking) can be found in Table 1.

**Table 1:** Sociodemographic characteristics of self-identified Indigenous individuals by cohort

Sociodemographic characteristic	Indigenous cohort	
	Comprehensive cohort	Tracking cohort
*N*	1128	751
Sex *n* (%)		
Male	497 (44.1)	328 (43.7)
Female	631 (55.9)	423 (56.3)
Age *n* (%)		
45–54	440 (39.)	285 (37.9)
55–64	376 (33.3)	266 (35.4)
65–74	217 (19.2)	132 (17.6)
75–85	95 (8.4)	68 (9.1)
Highest level of education *n* (%)		
University	369 (32.8)	169 (22.7)
Postsecondary	466 (41.5)	289 (38.8)
Secondary	289 (25.7)	286 (38.4)
Household income *n* (%)		
< 20,000	101 (9.4)	66 (9.4)
20,000–49,999	257 (24.0)	230 (32.8)
50,000–99,999	356 (33.3)	241 (34.3)
100,000–149,000	193 (18.0)	92 (13.1)
> 150,000	163 (15.2)	73 (10.4)
Marital status *n* (%)		
Single or never married	149 (13.2)	100 (13.3)
Married or common-law	698 (61.9)	492 (65.5)
Widowed	85 (7.5)	57 (7.6)
Divorced	140 (12.4)	85 (11.3)
Separated	55 (4.9)	17 (2.3)
Religion *n* (%)		
Roman Catholic	490 (43.6)	339 (45.2)
Other Christian	313 (27.8)	250 (33.4)
Other/multi-religion	73 (6.5)	27 (3.6)
No religion	249 (22.1)	133 (17.8)

Education: “University” education includes bachelor’s degree or above, “postsecondary” education includes college, CEGEP, apprenticeship, trades, or other non-university certificate or diploma

Baseline sociodemographic data of the entire Indigenous-identified cohort (comprehensive and tracking) and non-Indigenous cohort can be found in Table 2. Compared to the Indigenous cohort, the non-Indigenous cohort contained a higher proportion of individuals with university level education, a higher median age, and a lower proportion of individuals with a household income of less than $20,000.

**Table 2:** Sociodemographic characteristics of CLSA cohort by self-reported Indigenous status

Sociodemographic characteristic	Full cohort	
	Indigenous	Non-Indigenous
*N*	1879	48,935
Sex *n* (%)		
Male	825 (43.9)	24,110 (49.3)
Female	1054 (56.1)	24,825 (50.7)
Age *n* (%)		
45–54	725 (38.6)	12,520 (25.6)
55–64	642 (34.2)	15,594 (31.9)
65–74	349 (18.6)	11,556 (23.6)
75–85	163 (8.7)	9265 (18.9)
Highest level of education *n* (%)		
University	538 (28.6)	20,001 (49.9)
Postsecondary	755 (40.2)	16,203 (40.4)
Secondary	575 (30.6)	3674 (9.2)
Less than secondary	10 (0.5)	213 (0.5)
Household income *n* (%)		
< 20,000	167 (9.4)	2687 (5.9)
20,000–49,999	487 (27.5)	11,588 (25.3)
50,000–99,999	597 (33.7)	16,372 (35.8)
100,000–149,000	285 (16.1)	8378 (18.3)
> 150,000	236 (13.3)	6743 (14.7)
Marital status *n* (%)		
Single or never married	249 (13.3)	4054 (8.3)
Married or common-law	1190 (63.4)	33,708 (68.9)
Widowed	142 (7.6)	4983 (10.2)
Divorced	225 (12.0)	4898 (10.0)
Separated	72 (3.8)	1279 (2.6)
Religion *n* (%)		
Roman Catholic	829 (44.2)	16,798 (34.5)
Other Christian	563 (30.0)	19,614 (40.3)
Other/multi-religion	100 (5.3)	1983 (4.1)
No religion	382 (20.4)	10,298 (21.2)

Education: “University” education includes bachelor’s degree or above, “postsecondary” education includescollege, CEGEP, apprenticeship, trades, or other non-university certificate or diploma

## Discussion

Though it was not the intent nor design of the CLSA to collect and develop an Indigenous-identified cohort, the baseline data collection included information regarding Indigenous identity. Given that the CLSA contains Indigenous-identified data, Indigenous-led, ethical, and appropriate data analysis policies are warranted. This work was initiated by the Native Women’s Association of Canada to develop a comprehensive, community-driven profile of healthy aging and health system use in Indigenous communities across Canada. Unfortunately, the CLSA dataset proved unable to provide such a profile. Our interpretation of this self-identified Indigenous sample in the CLSA cohort was guided by an Elder Advisory Circle who raised important concerns with (1) lack of Indigenous data governance in CLSA processes, (2) lack of Indigenous perspectives and experiences in the survey content, and (3) non-representative sampling of Indigenous populations.

This analysis revealed important sampling considerations for the Indigenous-identified cohort. The relatively small national sample of Indigenous older adults included in the CLSA is limited not only in numbers (3.7% of national sample) but also in representation. The majority (60%) of Indigenous-identified survey respondents belonged to the comprehensive cohort, which recruited from a 25–50-km sampling radius around 11 urban study sites. This resulted in an urban-centric sampling approach and limited sampling opportunities to only those living off-reserve. According to the 2021 Canadian Census, approximately 82% of the Indigenous population reported living off-reserve (Statistics Canada, 2023); however, it is likely that many Indigenous people living off-reserve reside beyond the sampling radius of the urban study sites. Beyond this, limiting the language of assessment to only English and French further impacted participation opportunities for community-dwelling non-English/French-speaking older Indigenous adults, likely contributing to overall selection bias.

The Elder Advisory Circle maintained suspicion and concern with the representativeness of the sample of self-identified Indigenous people. For example, one point of contention was the percent of respondents who identified as Catholic (44.2%). The Elder Advisory Circle felt that this value was higher than expected, especially given that spirituality was not included in the survey. The proportion of Indigenous individuals who identified as Catholic was also ~ 17% higher than reported in the Indigenous Population Profile (off-reserve) from the 2021 Census (S. Canada, 2023). The Indigenous sample in the CLSA cohort was also highly educated, with 28.6% completing university and 40.2% completing postsecondary education. The proportion of individuals with education beyond high school (secondary) in this CLSA cohort far exceeded the educational attainment in the Indigenous Population Profile (off-reserve) from the 2021 Census, which reported 8.6% completed university and 32.4% postsecondary education (Statistics Canada, 2023).

The resulting limited sample size and approach did not allow for a distinctions-based approach both in differentiating between First Nations, Inuit, and Métis and within these groups (i.e., on-reserve vs. off-reserve). Consequently, a pan-Indigenous approach to the analysis was taken. Such an approach perpetuates historical misclassification/categorization of Indigenous identity (Madden et al., 2019) and does not respect and acknowledge these distinct Peoples with unique cultures, histories, rights, laws, and governments. In the future, data systems must utilize distinctions-based approaches, including nation-based approaches, which recognize the unique histories, jurisdictions, and realities within and between First Nations, Inuit, and Métis (Smylie et al., 2020).

Given both the sampling and selection bias and the concerned responses from the Elder Advisory Circle, it is a reasonable conclusion that neither the tracking nor comprehensive cohort adequately reflects the realities of Indigenous people living off-reserve. This analysis was conducted alongside Indigenous partners including NWAC and the Elder Advisory Circle. The voices of these Indigenous partners guided all aspects of this work, but were not analyzed in and of themselves. For example, feedback from the Elder Advisory Circle informed the interpretation of this analysis, though no formal interviews were conducted or included.

Achieving a deeper understanding of the limitations of the data is not without merit. Ultimately, the more work that can be done to understand the limitations of the data, the better equipped we will be to determine how the data could or should be used — if at all; however, this determination must be directed and led by Indigenous Peoples. Indigenous Data Sovereignty maintains that Indigenous Peoples hold authority over data about their nations, citizens, communities, and non-human relations, regardless of the location of those data (Carroll et al., 2022; Kukutai & Taylor, 2016; Rainie et al., 2019). Principles of Indigenous Data Sovereignty include that Indigenous Peoples have the power to determine who is counted among them, not only dictating the content and approach of data collected about them but also retaining the power to determine who can access that data (Gray et al., 2024; Walker et al., 2017). Health research organizations and service providers that routinely collect Indigenous-identified data are ethically obligated to build meaningful and formal partnerships that recognize Indigenous Sovereignty with respect to their data (Gray et al., 2024; Rowe et al., 2021; Steffler, 2016).

A critical component of Indigenous-led research — particularly within the topic of healthy aging — is community engagement and integration of traditional knowledge. Elders and older Indigenous people play a vital role in the balance, health, and well-being of their communities (Turcotte & Schellenberg, 2007). Older Indigenous people’s health and well-being is directly linked to healthy, thriving, vibrant Indigenous communities and cultures (Turcotte & Schellenberg, 2007). Older Indigenous people are crucial in preserving and passing down languages, teachings, and traditions to the next generation (Turcotte & Schellenberg, 2007). Efforts that work to ensure Elders and older Indigenous people are able to successfully age in ways that are culturally safe, community based, and reflective of their unique needs and priorities are an essential part of the reconciliation process.

## Conclusion

Community engagement was integral to this study such that community partners co-developed the analysis plan and collaborated on the analysis and interpretation of results, which was guided by the input of the Elder Advisory Circle. Our engagement with the Elder Advisory Circle and the comparison to other Indigenous data (including but not limited to the Canadian Community Health Survey) have led us to recognize that the CLSA data may not fully capture the nuanced sociodemographic profile of older Indigenous Peoples. Consequently, we advise exercising great caution when using these data. It is important to note that any comparisons involving non-Indigenous subsample or other subgroups may be subject to bias, as these data may not comprehensively reflect the diverse realities of Indigenous Peoples in Canada.

The CLSA has acknowledged these findings and has fervently committed to addressing these concerns. The longitudinal nature of the study does not allow for responsive revisions to the sampling approach; however, there remain opportunities for improvement in both culturally relevant survey content and Indigenous data governance. For example, CLSA is initiating a working group to inform and assess existing survey indicators and inform future data collection. To address concerns around the governance and use of existing Indigenous data in the CLSA, the CLSA consulted further with Indigenous researchers and partners beginning in 2019 to revise its data access process and ensure any subsequent analysis involving First Nations, Métis, and Inuit is respectful of Indigenous data governance principles. The revised data access policy requires data requestors who are requesting Indigenous identifiers to describe their engagement with Indigenous Peoples, as required by Articles 9.1 and 9.2 of the Tri-Council Policy Statement: Ethical Conduct for Research Involving Humans. The revised policy is supported by new internal processes to align the CLSA and its Data and Sample Access Committee with the new requirements. This collaborative modification to the CLSA process at the direction of Indigenous partners and researchers is a concrete step towards reconciliation and honouring the data rights of Indigenous Peoples.

Historically, non-Indigenous researchers have often carried out research and subsequent analyses with Indigenous data that are not contextualized or relevant to Indigenous communities (Drawson et al., 2017). Guaranteeing Indigenous Peoples have self-determination over Indigenous research and, subsequently, how the results are analyzed and used is not only essential to ensuring effective research and policies (Hyett et al., 2019), but it is also a legal imperative as outlined in the UNDRIP (United Nations, 2007) which affirms the rights of Indigenous Peoples to self-determination and to be able to control and protect their traditional knowledge. There remain opportunities for other administrative health databases that contain Indigenous-identified data to revise data access and analysis processes to align with Indigenous data governance principles.

## Contributions to knowledge

What does this study add to existing knowledge?

- This study uses Indigenous methodologies for quantitative methods to conduct a community-engaged analysis of quantitative data.
- Though not originally intended nor designed to be an Indigenous-specific cohort, the baseline data collection for the Canadian Longitudinal Study on Aging (CLSA) did include some information regarding Indigenous identity. This study sheds light on the intricacies of this self-identified, Indigenous sample, recognizing that it emerged unintentionally.

What are the key implications for public health interventions, practice, or policy?

- This study provides recommendations for the appropriate and respectful use of the Indigenous-identified data in the CLSA.
- The findings have already informed policy with a fervent commitment by the CLSA to improve survey content to be more culturally relevant and to establish Indigenous data governance.

## Data Availability

Data are available from the Canadian Longitudinal Study on Aging (www.clsa-elcv.ca) for researchers who meet the criteria for access to de-identified CLSA data.

## Declarations

### Disclaimer

The opinions expressed in this manuscript are the author’s own and do not reflect the views of the Canadian Longitudinal Study on Aging.
